# A New Technique for Placement of Blocking Screws and its Mechanical Effect on Stability of Tibia Fractures with Distal Fragments after Insertion of Small‐Diameter Intramedullary Nails

**DOI:** 10.1111/os.13149

**Published:** 2021-10-01

**Authors:** Cong‐ming Zhang, Liang Sun, Qian Wang, Qiang Huang, Hua Lin, Ning Duan, Chen‐chen Zhang, Teng Ma, Han‐zhong Xue, Kun Zhang, Zhong Li

**Affiliations:** ^1^ Department of Orthopaedics and Traumatology Xi'an Jiaotong University College of Medicine, Hong‐Hui Hospital Xi'an China

**Keywords:** Biomechanical evaluation, Distal tibial fractures, Novel blocking screw, Small‐diameter intramedullary nailing, Unstable fractures

## Abstract

**Objective:**

To design a novel blocking screws (BSs) geometry and insertion method to treat distal tibia fracture with nailing and comparison of mechanical properties of novel and traditional screws.

**Methods:**

Twenty‐one synthetic left tibiae were sectioned to obtain 21 distal segments measuring 55 mm. Intramedullary (IM) 9‐mm tibial nails were advanced to 6 mm from the ankle joint. Two transverse and one anterior–posterior (AP) locking screws were inserted. Both medial–lateral (ML) BSs were placed 10 mm from the topmost interlocking screw. A custom‐made jig assisted in placing the novel and traditional BSs. The time spent in placing each BS was recorded. All the samples were repaired with an IM nail and without BSs, with two traditional BSs, and with two novel BSs. An initial loading from −150 to +150 N was applied to specimens in the ML direction at 185 mm from the nail end, followed by cyclic loading of the same for 10,000 cycles with failure‐to‐test loading of 350 N in the ML direction. The maximum displacement was measured at 80 mm from the nail end and recorded under initial loading. The damage of two kinds of BSs to the nail was recorded.

**Results:**

Compared with average 5.21 min of the time of placing a traditional BS, the time spent in positioning a novel BS on the fracture model was 2.53 min. In the distal bone–implant constructs (BICs), the addition of traditional BSs decreased the maximum displacement of the BICs by 26.2%. The addition of the novel BSs decreased the displacement by 28.9%. All constructs survived 10,000 cycles without hardware deformation. The failure rate of the control group was significantly greater than that of the traditional group; however, the novel group was similar to the traditional group. The damage of the traditional BS to the nail was greater than that of the novel one.

**Conclusions:**

The novel and traditional BSs are comparably effective for increasing the primary mechanical stability of distal metaphyseal fractures after nailin. However, compared to the placement of a traditional BS, implanting a novel BS took more less time and caused less damage to the nail. Additionally, the most obvious advantage of the novel BS design and insertion technology was that the pressure and distance between it and the IM nail could be controlled by rotating the screw. These advantages of the novel BS will be beneficial for clinical application.

## Graphical abstract

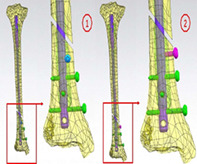

This study introduces a new blocking screws (BSs) structure and compares the mechanical stiffnesses of novel and traditional BSs using a distal tibial fracture segment model treated with intramedullary nails. the most obvious advantage of the novel BS design and insertion technology was that the pressure and distance between it and the IM nail could be controlled by rotating the screw, which helped to shorten the adjustment time and reduce the occurrence of new fractures. By mechanical test, this study concluded that the stiffnesses of the novel and traditional BSs are equal.

## Introduction

Distal tibia fractures are treated by various methods such as intramedullary (IM) nailing, plating, and external fixation, but an optimal treatment technique has not yet been established for clinical application^1^. Application of the IM nailing method decreases the risk of soft tissue complications compared with the use of the plate fixation method^2^, ^3^. However, the reported rate of post‐operational malalignment during IM nailing has reached 14%–23%^4^, ^5^, ^6^. This complication is often followed by the insufficient mechanical stability of the IM nails, owing to distal widening of the medullary canal and the low support strength of the small‐diameter tibial IM nail^4^, ^5^, ^6^. To improve the effectiveness of IM nailing for treating distal tibial fracture, various techniques such as external fixation^7^, fibular fixing with a plate^8^ to hold the alignment, and assistant nail fixation have been employed. Another common tool that is used to improve the reduction and fixation is a blocking screw (BS)^9^.

Krettek *et al*. were the first to propose the use of a BS to assist the fixation of metaphyseal fractures for increased bone‐nail stiffness^10^. By narrowing the medullary canal in the metaphyseal or flared segment of the bone, a BS enables the stability of bone‐nail constructs. In a mechanical study, Krettek *et al*.^10^ reported that the addition of BSs in the proximal tibial fracture model reduced the displacement of the bone‐nail complex by 25% under the ML direction of loading. In the distal tibial fractures, addition ML BSs increased the stiffness of bone–nail constructs by 57%. Their results demonstrated that in order to increase the stability of the bone–nail constructure, the placement of the BS should be as close to the fracture site as possible. BSs are predominantly used for femur and tibia fractures at the metaphyseal–diaphyseal junction to assist fracture reduction and stabilize the bone–implant construct through the provision of a third fixation point^9^, ^10^, ^11^, ^12^, ^13^, ^14^. A systematic review containing 13 studies with a total of 371 participants and 376 fractures showed that, compared with nailing alone, IM nailing with a BS has lower rates of nonunion and coronal malalignment when treating metaphyseal fractures^15^. Meanwhile, additional BSs can also decrease tibial callus formation^16^ owing to increased bone‐nail construct stability while treating the delayed union of proximal tibial shaft fractures *via* nailing^17^. These important advantages mainly depend on accurate BS positioning^10^, ^12^, ^13^, ^14^, ^15^, ^16^, ^17^, ^18^. Accurate placement also enables three‐point fixation principles, which help to overcome the mismatch of bone and nail at the diaphyseal‐metaphyseal junction or the metaphyseal that is responsible for the associated axial displacement.

Studies have been conducted to investigate the proper placement of BSs, such as at an acute angle to the flared segment between the long axis of the displaced fracture fragments and aligned with the plane of the fracture^19^, the opposite side of the thumbs^20^, and the pre‐use of a Steinmann pin^21^. However, these studies only highlighted the area of BS positioning. The concrete point of BS placement still relies on a surgeon's experience alongside x‐ray fluoroscopy during operation. In short, it is very difficult to implant a satisfactory BS using current techniques and methods. To enable the BS to provide accurate reduction and stability, it is often necessary to adjust its position repeatedly during the operation. However, multiple freehand adjustments prolong the operative time and increase the risk of nail damage, bleeding, loss of reduction, infection, and even new fractures^22^. The adjusting technique of BSs is therefore essential for achieving the maximum benefit of their use and requires an effective adjustment strategy. Hence, this important limitation of the operative tuning of traditional BSs requires improvements to simplify their clinical applications.

The first objective of this study is to describe the geometric construct of a novel BS. The novel BS geometry is very simple and improves the traditional BS screw tip by cutting it into a flat end. The second objective of this study is to introduce the placement method of a novel BS to improve its clinical application. The placement is parallel to the deformity of the fracture in a concave plane unlike the traditional perpendicular method. The point connecting with the nail of the flat end is its lateral plane. BS positioning adjustments can then be obtained by turning the BS instead of replacing it. This thread‐controlling adjustment strategy for metaphyseal fractures makes BS adjusting quite easy and avoids the need to accurately determine the entrance of the BS while avoiding additional injuries to soft tissues. However, it is unclear whether the mechanical property of the novel BS satisfies the need to enhance the BICs stability. Hence, the third objective of this study is to compare the mechanical stiffnesses of the two methods to supplement distal tibial metaphyseal fractures using small‐diameter IM nail fixations. Our null hypothesis states that the mechanical properties of additional BSs will be better than no BS; however, there will be no differences between the novel and traditional groups.

## Materials and Methods

### Materials and Groups

**Fig. 1:**
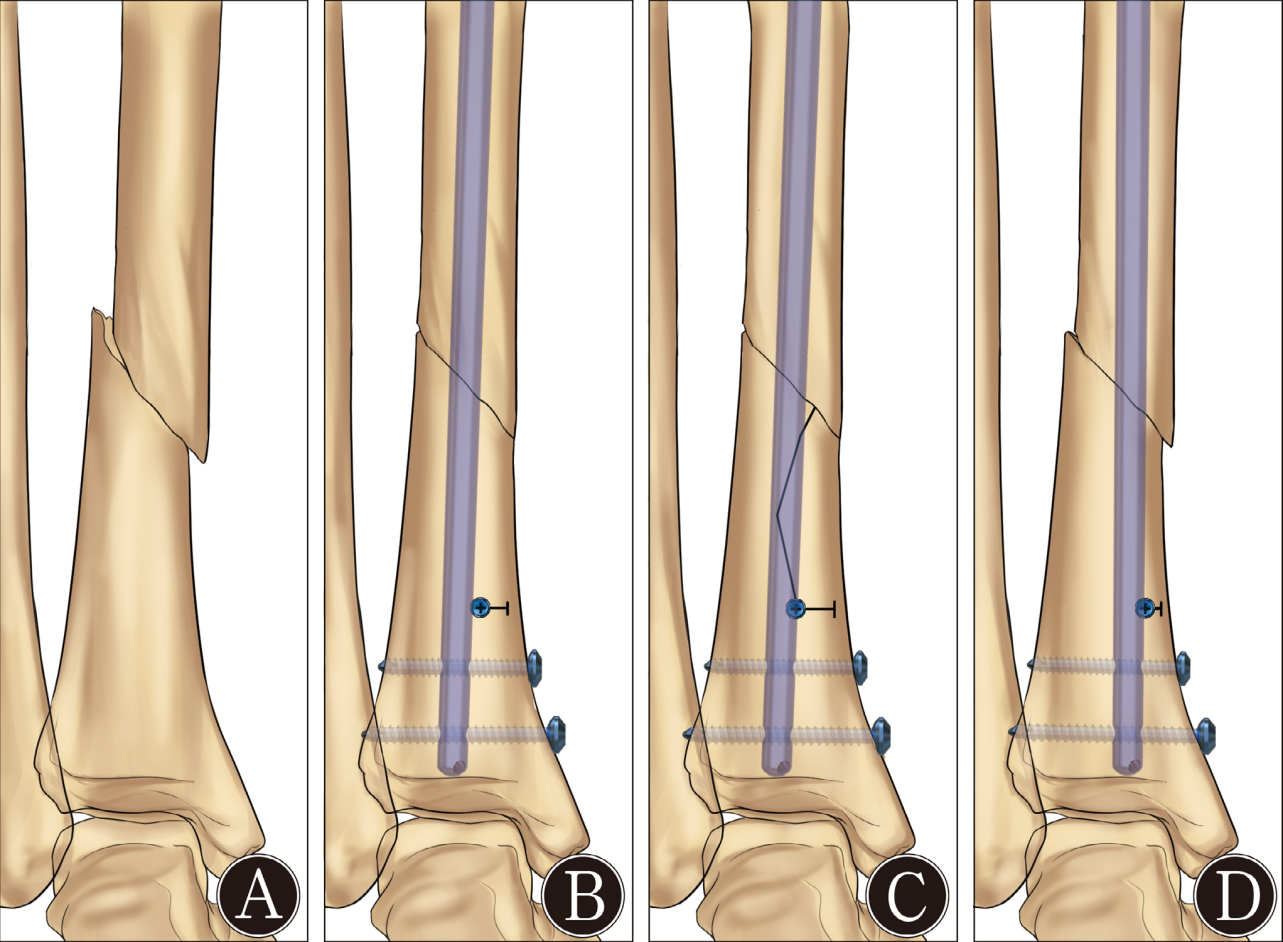
(A) Position of a traditional blocking screw, and (B) the modified position of the novel blocking screw.

A synthetic tissue surrogate with identical geometry and homogeneous material properties was selected. Twenty‐one (*n* = 21) fourth‐generation composite Sawbones left tibiae with solid cancellous foam (Model 3401; Pacific Research Laboratories, Vashon, WA, USA) and an expert tibial nail (nail diameter = 9 mm; IRENE, Tianjin, China) were used for the investigation. Previous studies have confirmed that, compared with human bone, surrogates produce remarkable results for axial, compression, torsional, and bending stiffnesses, as well as for failure mechanisms under different loading conditions^23^, ^24^, ^25^, ^26^, ^27^, ^28^. Three 4.2‐mm‐diameter bicortical locking screws, used in all specimens, were combined with two 3.5‐mm‐diameter cortical screws that were employed as BSs in seven (*n* = 7) tibiae per treatment group. The treatment groups were constructs without any BS except for the pre‐planned screw path (control group), those with two bicortical traditional BSs placed in the anteroposterior (AP) position (traditional group) (Fig. 1A), and those with two semi‐cortical novel BSs placed in the ML direction (novel group) (Fig. 1B).

### Fracture Model and Instrumentation

**Fig. 2:**
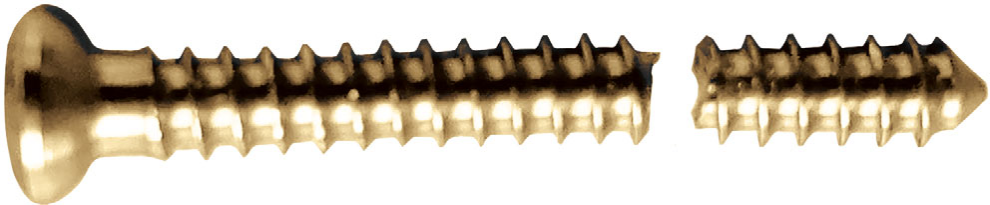
Distal segments measuring 55 mm were sectioned in all the specimens. A medial and a lateral blocking screw in the anterior–posterior direction were placed 10 mm above the superior‐most interlocking screw and 12 mm distal to the lower end of a segment.

**Fig. 3:**
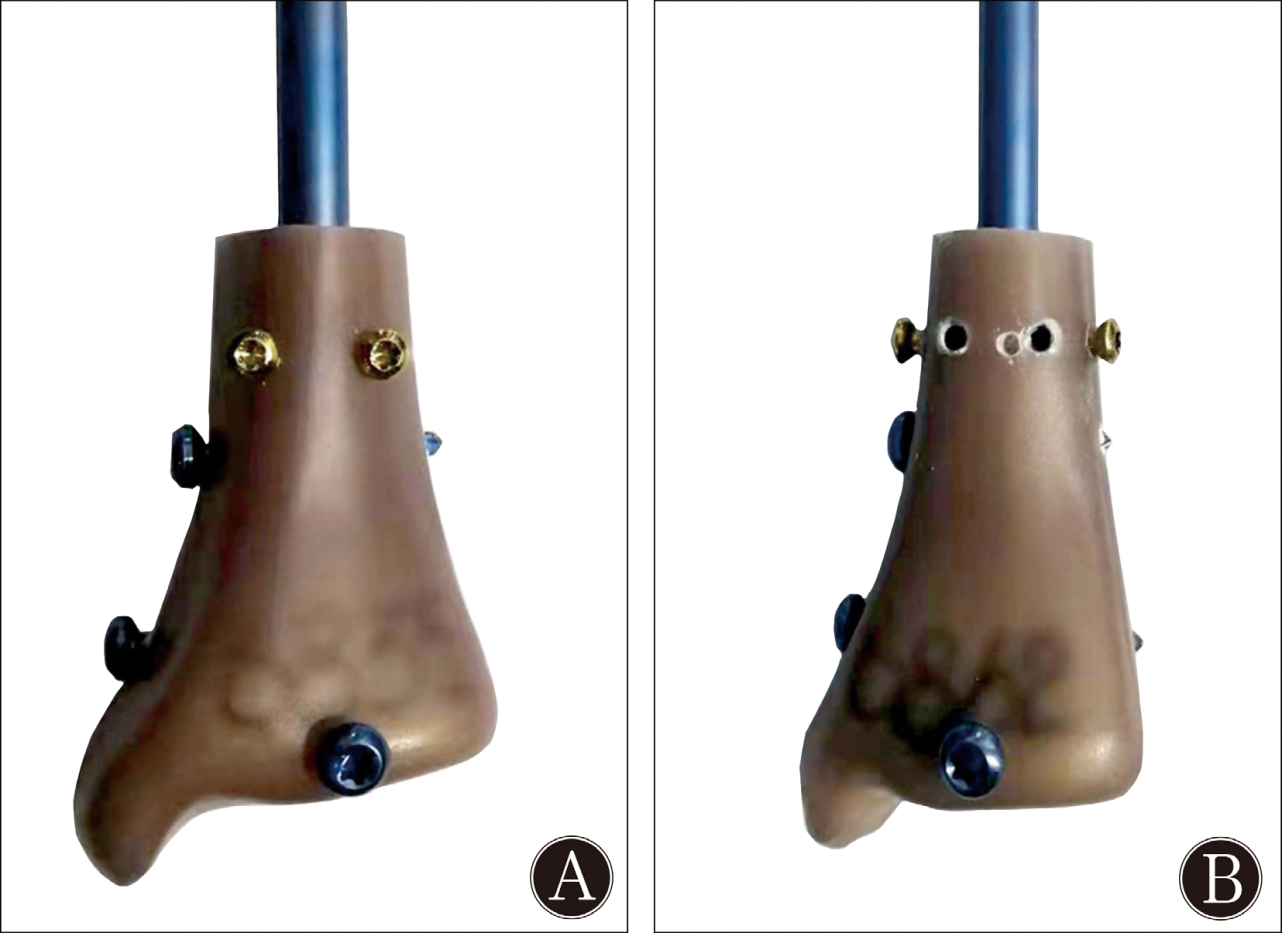
(A) Placement and touching point with the nail of the traditional blocking screw, and (B) strategy of placing a novel blocking screw and touching part of it.

**Fig. 4:**
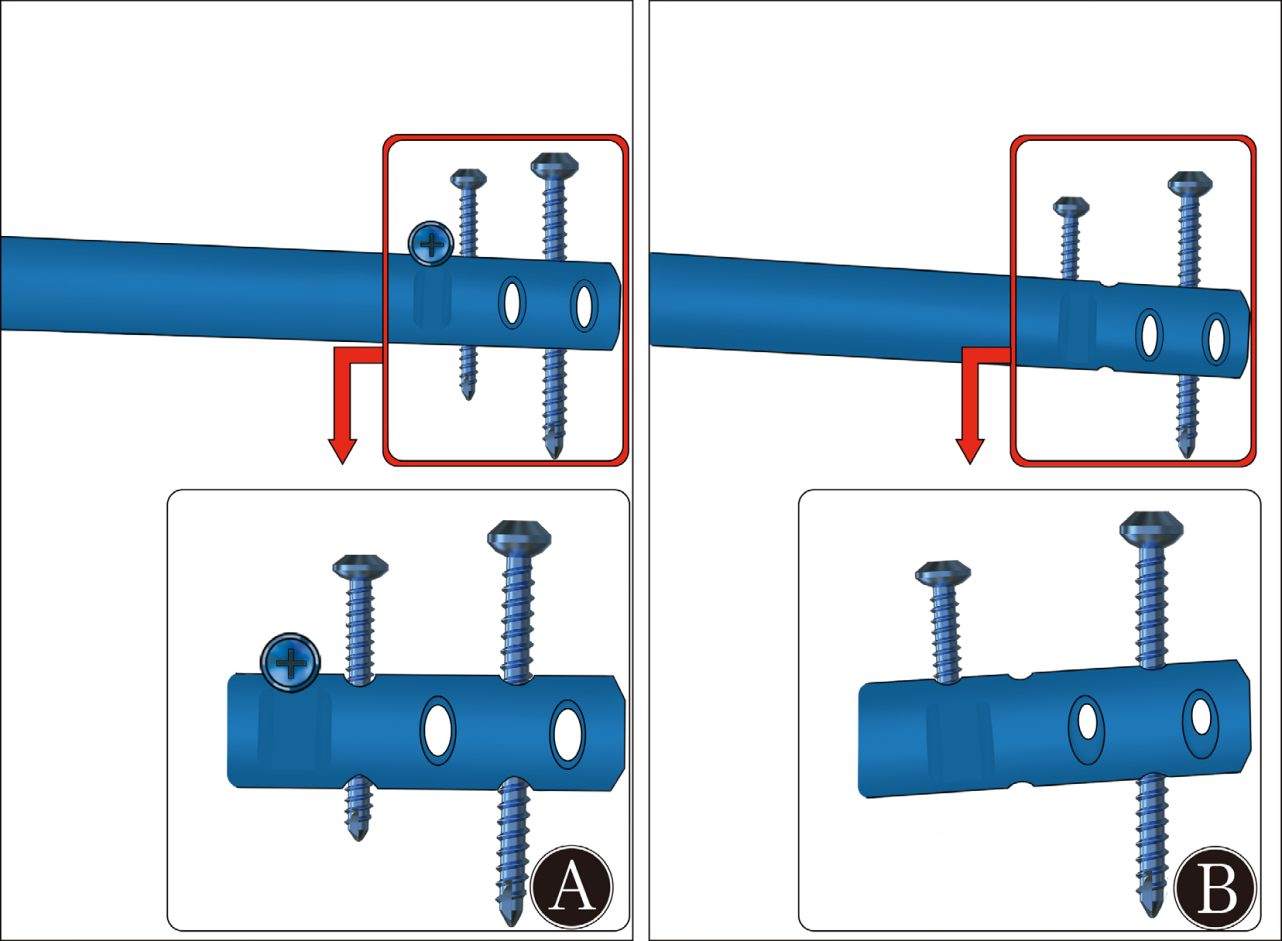
Novel BS made from a 3.5‐mm cortical screw with flattened tip.

The 9‐mm IM nail was inserted in an unreamed fashion using a standard technique^29^. An unstable distal tibial fracture was simulated by cutting the distal tibial segments at a distance of 55 mm from the tibial plafond in all specimens. The solid tibial nails were advanced to a point 6 mm from the ankle joint. The BSs were placed approximately 10 mm from the most proximal locking screw holes in accordance with the work of Krettek *et al*.^10^ (Fig. 2). The large difference between the diameters of the implant and the medullary cavity of the metaphysis was simulated in our model. Two ML BSs were inserted to avoid fracture displacement in the frontal plane. For the traditional BS, a bicortical hole was drilled with a 2.5‐mm bit using a custom‐made jig. A fully threaded 3.5‐mm cortical screw was placed on a two‐sided nail 10 mm from the proximal inter‐locking screw in the AP direction (Fig. 3A). The novel BS was made from a 3.5‐mm fully cortical threaded screw. The tail end of the 3.5‐mm cortical screw was cut and ground to a flat surface (Fig. 4). The retaining length of the new BSs was determined based on the distance between the outer cortex and the surface of the nail. The location was aligned to the connective line between the two ends of the distal traverse locking screws at 10 mm from the proximal locking screw. The outer cortex was drilled using a 2.5‐mm bit. Two novel BSs were placed on the ML side of the nail with the assistance of a custom‐made jig. When the end of the novel BSs touched the nail (Fig. 3B), they were further tightened with a screwdriver for half a unit circle to increase pressure between the nail and the BSs. Thus, the novel BS resulted in a modified tuning technique for positioning. The time required to place every BS was recorded. The consumed time of placing a BS was compared between the two groups, and the marker left on the nail by the two placement methods was recorded. The distal BICs were then embedded in the bone cement in a cast frame.

### Mechanical Testing

Loads ranging between −150 and +150 N (one‐third of the body weight of a 45.9‐kg person) were applied in the ML direction at 185 mm from the nail end after BIC fixation in a material testing machine^10^. Using a laser distance sensor, the maximum displacement was determined to be 80 mm from the end of the nail. The average maximum deformation and standard deviation were then calculated according to the method of Krettek *et al*.^10^. Assessments were performed by a senior orthopedic surgeon and the present author. The constructs that survived initial loading were then tested under cyclical loading. The instrumented constructs were then fixed laterally onto the pole of the load frame (MTS Mini Bionix.II, Model 359, MTS Systems Corp, Eden Prairie, MN, USA) using custom‐designed fixtures. Another custom‐designed fixture was rigidly installed to each nail at 185 mm from its end. Through this fixture, the nail was coupled to the actuator under the condition of the axis of the nail being parallel to the horizontal plane, which ensured that the direction of loading was ML. A loading from −150 to +150 N was then applied at a frequency of 1 Hz for 10,000 cycles. The BICs that tolerated cyclical testing were finally loaded at failure loading of 350 N (body weight of a 35.7‐kg person) in the ML direction.

### Research Indexes

#### Maximum Displacement

The maximum displacement was used to evaluate the stiffnesses of the BICs. The BIC stiffness is an interesting mechanical parameter and is defined as the slope of the force *versus* displacement curve. Under the same loading, the smaller was the maximum displacement, the greater was the stiffness.

#### Cyclical Loading and Failure to Test

**Fig. 5:**
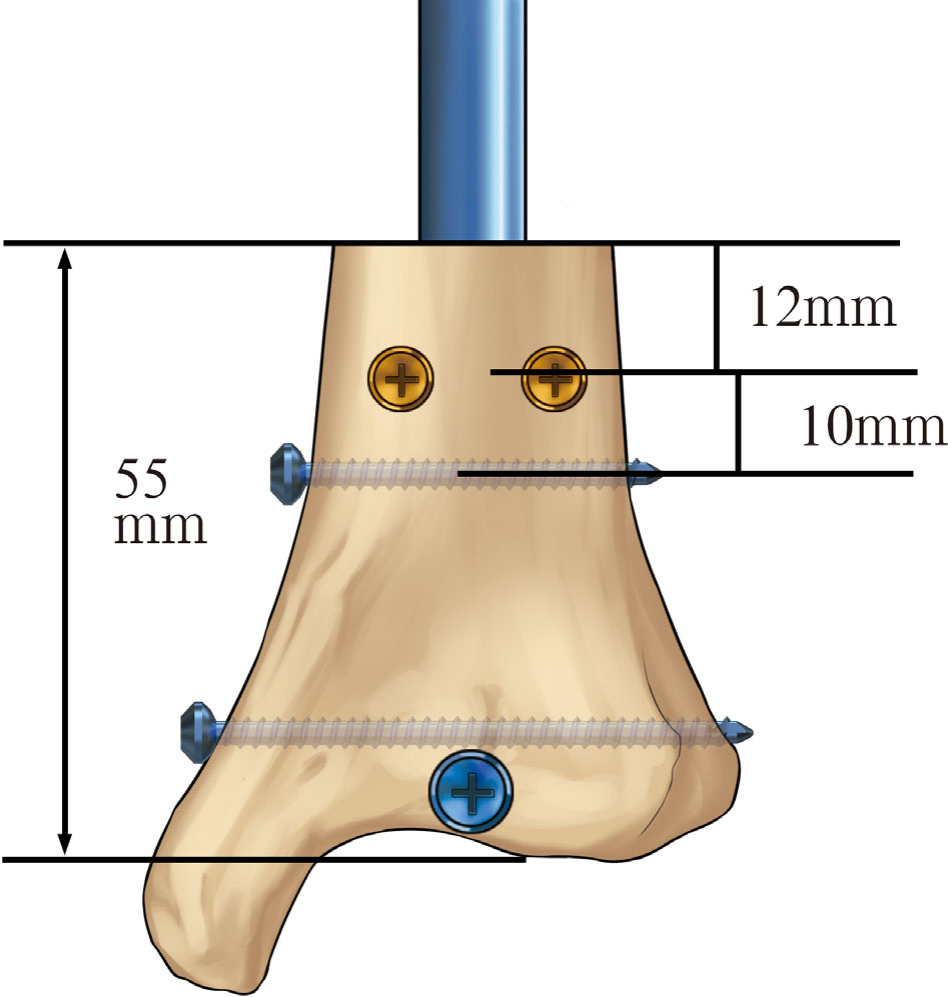
Representative images of various failures noted from testing: (A) distal vertical bone fracture with a screw hole in the novel group; (B) distal transverse bone fracture in the interface between bone‐implant constructs and bone cement in the novel group; (C) serious nail loosening in the control group.

As mentioned earlier, loading from −150 to +150 N was then applied at a frequency of 1 Hz for 10,000 cycles in the ML direction, which represents the approximate number of steps taken over a 4–6 weeks period; i.e., the estimated interval for postoperative non‐weight bearing^30^. This test refers to the method of Hoenig *et al*.^31^ and was used to evaluate results of the fatigue test of BICs. To our knowledge, there is no data reference regarding the failure to test of a biomechanical experiment in the ML direction. The data of 350‐N loading was therefore based on the failure of more than half of the samples in the control group from our pre‐experiment. Failure was defined as catastrophic, manifesting as a bone fracture, loosened nail or bending, or other gross hardware breakage (Fig. 5).

#### Time Spent in Placing a BS

We used the consumed time to compare the difficulty of placing a respective traditional and novel BS. The consumed time is an indirect index to evaluate the possible subsequent surgical complications.

#### Damage to a Nail

The damage to a nail is defined as the mark left on a nail by the friction of the drilling head and cutting of the screw thread. This indicator was used to evaluate the destruction to a nail when placing a traditional or novel BS.

### Statistical Analysis

All data were initially tested for normality distribution using the Shapiro–Wilk statistical test. One‐way ANOVA tests (two‐tailed) and a least‐significant‐difference post‐hoc test were used to compare the results within the group. The measurements included time spent in placing a BS and displacement of the nail. Two independent sample *t*‐tests were used to compare the placing time of the novel and traditional BSs. The difference was considered to be significant when the *P* value was less than 0.05.

## Results

### Maximum Displacement of Nail under Transverse Loading From −150 to +150 N

In the distal BICs, the addition of traditional BSs decreased the maximum displacement of the BIC by 26.2%, from 4.88 ± 1.20 mm (mean ± standard deviation) in the control group to 3.60 ± 0.72 mm in the traditional BS group (*F* = 5.004, *P* = 0.018). Compared with 4.88 ± 1.20 mm (mean ± standard deviation) in the control group, the maximum displacement of the novel group was 3.47 ± 0.77 mm, which decreased by 28.9% (*F* = 5.004, *P* = 0.010). A comparison of the traditional and novel groups showed that they were not statistically significant. The increased stability of BIC in the novel BS was more than 2.7% greater than that of the traditional group, although no significant difference was observed.

### Cyclical Loading and Failure to Test

All specimens in the three groups survived initial and cyclic loading. Under a loading of 350 N, failure in the control group resulted in the nail seriously loosening in two specimens, one fracture, and nail bending in two specimens. Two of the seven specimens in the traditional group failed and distributed into a new fracture. The incidence rate and distribution of failures in the novel group was similar to that of the traditional group (Fig. 5). The breakage of the nail and locking screw was not found until all experimental procedures were complete. In all experimental groups, especially the novel group, no backout or breakage of the BS was found. Interestingly, new fractures mainly occurred along the interface between the BICs and bone cement, and four of five new fractures occurred in all groups. All nail loosening and bending occurred in the control group.

### Time Spent in Placing a BS

The average time needed to place a single BS on the fracture model in the new BS group was 2.53 min (range: 1.8–3.2 min), which was less than 5.12 min (range: 4.2–6.1 min) in the traditional group (*t* = −7.798, *P* < 0.001). Time spent in the placement of the traditional BS required nearly twice as much time as the novel group.

### Damage to the Nail When Inserting BS

**Fig. 6:**
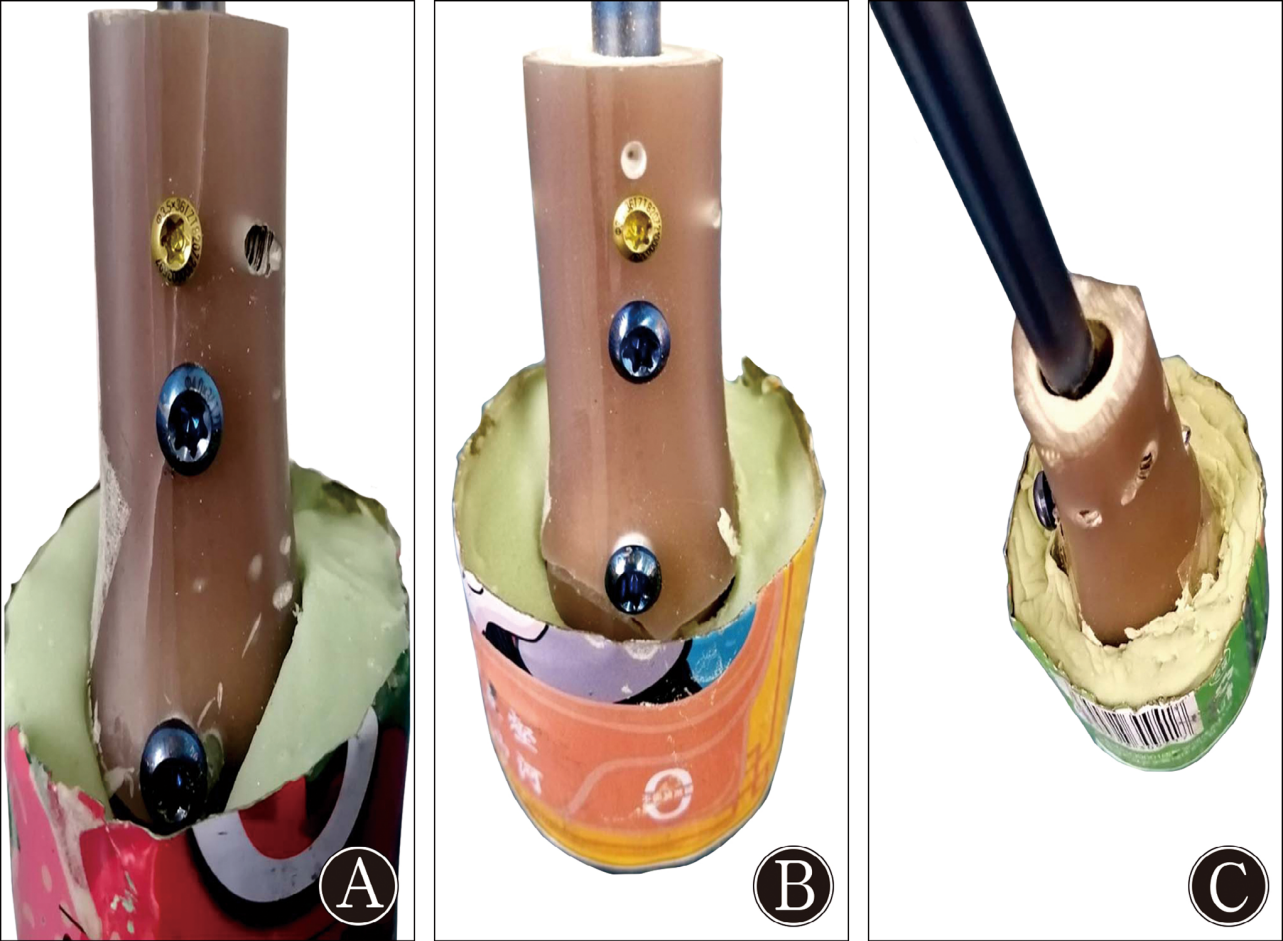
(A) Subtle scratch was shown on the nail in the novel group, and (B) deeper thread‐cutting marks were observed in the traditional group.

Slight damage occurred at the contact part between the BS and the nail in the novel BS group (Fig. 6A); however, more serious damage occurred in the traditional group (Fig. 6B). A longer time required by the drill bit to drive the nail and the cortical screw in the traditional group was observed compared with the novel group, owing to the differences of insertion methods:

## Discussion

### Difference in the Method of Adjusting the Distance

**Fig. 7:**
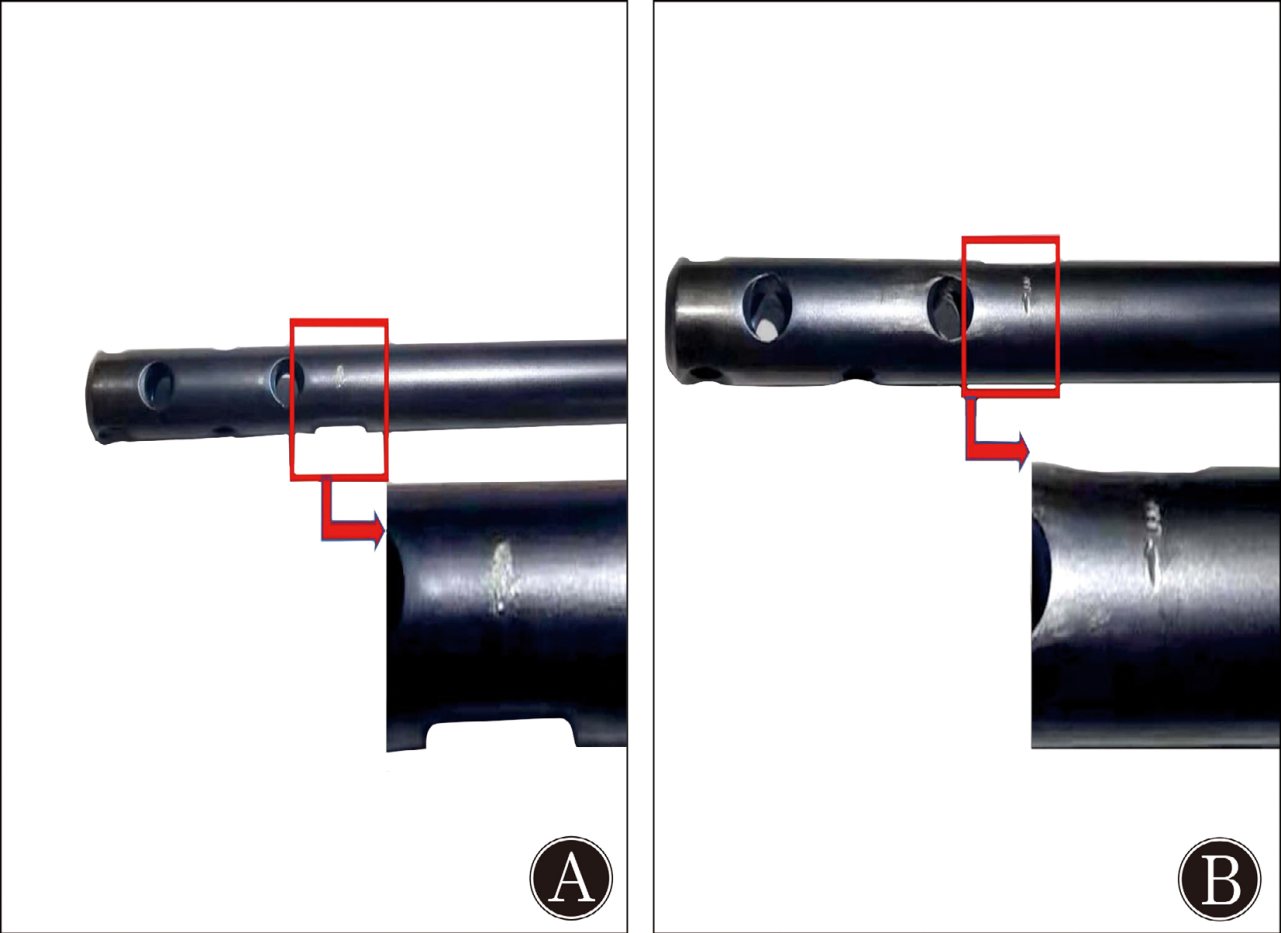
(A) Model with distal tibial fracture reduced and fixed with three different types of bone‐blocking screw–nail constructions (B–D). The positions of the blocking screws are indicated by the black points as follows: (B) when the position of a blocking screw was extremely close to the nail, fracture occurs; (C) the reduction was unsatisfactory when the distance between the blocking screw and the nail was large; (D) the reduction was perfect when the positions of the blocking screws were ideal.

The orientation of the traditional BS placement was perpendicular to the plane of the fracture deformity to enable the reduction and fixation of the flared segment. Thus, the effect of BS reduction and fixing the fracture segment was determined by its entry. The current difficulty involved the estimation of an appropriate entry point for placing the BSs. This endeavor remains a challenge for surgeons under conditions of skin shielding and fracture deformity. Although some precise methods for BS insertion have been described in recent literature^11^, ^13^, ^14^, ^19^, ^20^, ^21^, the extent to which they narrow the IM cavity *via* the BS depends mainly on the use of inter‐operational fluoroscopy and the experience of the surgeon. To obtain a perfect alignment, considerable time is required to adjust the location of the BS. Adjustments to the BS location during operation also prolong the operation time, resulting in bleeding and sometimes additional fracture lines^22^ (Fig. 7C). Nevertheless, modifying the positioning strategy of the traditional BS circumvents the clinical need to improve bone fracture healing prognosis. We herein described a novel BS geometry and its placing and adjusting method. The entrance of the novel BS does not need to be precisely located, and the distance between the novel BS and the nail can be controlled *via* screw‐turning. The placement of the novel BS is easier than that of the conventional method, and its placement will not lead to new fractures on account of its tuning method. To comprehensively understand the advantage of the novel BS, it was divided into an anchored end (the part touching the bone cortex) and a functional point (the part touching the nail).

The distance between the nail and the functional point was determined by the anchored end, resulting from the placement of the traditional BS perpendicular to the displacement of the flared segment. The tuning of the BS functional point was achieved *via* the movement of the anchored end. The current technique used to adjust the traditional BS requires resetting it, which requires even more time and consequential injury. To improve the placement and function of the BS, we modified its placement from a perpendicular position to the fragment movement plane parallel to it, thereby improving the placement and adjustment strategy. Meanwhile, the tip of the screw was designed to be flat to increase the connecting opportunity with the nail (Fig. 4). Koller *et al*. in 2020 described the clinical use of a fully threaded 3.5‐mm cancellous screw as a reduction tool for correcting frontal deformities along the coronal plane^32^. A reduction screw then was replaced with a traditional BS after the desired correction was obtained.

### Method of Placing

Our idea of using the BS as a reduction tool is similar to that of Koller *et al*.^32^, however, it differs in that the novel BS described herein acts as a stabilizing tool. It is placed adjacent to the nail without the need to replace it when the desired reduction is obtained. Before IM insertion, the novel BS can be inserted on the concave side of the deformity closer to the fracture in the coronal plane to reduce fracture segmentation, owing to the greater opportunity of the flat end of the BS to touch the nail. The novel BS can also be placed along the central axis of the IM parallel to the frontal locking screws or along the nail axis oriented as the frontal locking screws. This functions as a stability tool to enhance bone‐nail‐construct stiffness after nail insertion^10^. The key advantage of this novel BS is that the distance and compression between the nail and BS can be adjusted by turning the screw. Three effective clinical results are obtained through this advantage: the adjustment of the BS location does not need to be reset; nail damage coming from the drill bit and the BS thread is subtler; and secondary fractures caused by BS positioning all but disappear. However, the mechanical stiffness of the novel semi‐cortical BS is unclear, and we hypothesize that it is sufficient to ensure construct stability.

### Comparison of Mechanical Properties

This study demonstrates that both the novel transversal and traditional sagittal BSs, when placed adjacent to an IM nail, can increase the primary stability of distal tibia fractures. Using this model, the fracture was stabilized distally by two ML locking screws and one anterior–posterior locking screw. The fracture stiffness of the three locking screws was 26.2% less than that of the traditional BSs. The novel BSs increased construct stiffness up to 28.9%. Nevertheless, compared with the traditional group, no statistical differences were observed. The increased BIC stiffness of the novel BS was 2.7% greater than that of the traditional group. This could possibly lead to complaints from patients regarding the increased pressure resulting from the connection between the BS and nail. The traditional BS can only be used as an occupation screw to centralize the nail. However, the novel BS functions as a compression screw to reduce the nail and increase the construct stiffness. Breakage or backout of the novel screws was not observed until the end of each sample test. This may be explained by the fact that the force between the BS and the nail is less than the anti‐pull‐out strength of the interface between the bone and the semi‐cortical screws under a 150‐N load. In 2019, Ketata *et al*.^33^ used a finite element model of the synthetic bone by computed tomography scanning to test the anti‐pull‐out strength of the 4.5‐mm semi‐cortical bone screw. It was determined to be 439 N. This finding is in relation to 4.5‐mm screws, indicating that the anti‐pull‐out strength of 3.5‐mm semi‐cortical screws can satisfy the mechanical requirements of the BS. Under the same experimental conditions, Krettek *et al*.^10^ validated that the BIC stability could be increased by 57% by placing additional sagittal two‐sided BSs, showing more effective results than those of this study. The prior study utilized human cadaveric tibiae and a stainless‐steel tibial nail, which may be a critical reason for the difference.

The 350‐N loading‐failure test resulted in the only new fracture, whereas the control group resulted in considerable nail bending and loosening (Fig. 5C). One impossible explanation is that the increased stiffness of the BICs caused by the additional BS led to the new fracture occurring on the interface between the BIC and bone cement (Fig. 5B), owing to the relatively larger movement of this interface. No BS backout occurred in the novel group for both cyclical loading and intact failure testing, which provides more evidence that the anti‐pull‐out force of a 3.5‐mm semi‐cortical screw can satisfy the clinical need.

### Comparison of the Time Required

The time required for novel BS insertion required less than 50% of that of the traditional BS. Clinically, the insertion of a traditional BS may require more time on account of the existence of complex soft tissues, fracture displacements, and the lack of precise markers and assistive instruments. Compared with the traditional BS, inserting the new one was easier owing to the use of interlocking as an obvious marker, the tuning of the BS location achieved by turning the screw, and the lack of any need to precisely position it. Moreover, the continuous friction against the nail when using the traditional BS caused damage because of threading and drill cutting. However, the contact between the novel BS and the nail was transient, and only miniscule damage was found in the novel BS group.

### Reason Why Only the ML BS Mechanical Properties are Tested

With most tibial nails, two transverse locking screws are implanted to provide stability in the sagittal plane after reduction. However, these locking screws provide less stability in the frontal plane. Therefore, the direction of fracture translation is often along the axis of the locking nail in the frontal plane. For this reason, our model simulated the direction of fracture re‐displacement as an ML movement on the distal tibia fracture, which was fixed by nailing it with two transverse and one AP locking screws. Additional BSs were placed on the ML side to avoid translation of the ML interlocking screws. Additionally, the direction of the loading application was in the ML orientation.

### Limitations

This study had several limitations. Only ML testing was conducted on the current transverse fracture model. Regarding the novel method for placing BSs, further study is needed to quantify the effects of axial fatigue loading. However, a prior biomechanical study confirmed that a single additional medial BS has no effect on the distal tibia fracture model when nailing with the BS under combined cyclic axial and torsional loads. Additionally, compared with the two distal locking screws, three have significant advantages^34^. Another previous study^35^ used finite element analysis to confirm that BS application has no additional effect on the distal tibia fracture model through a comparison of fixation with plating and nailing with the BS. The rigidity of the bone–nail construct depends mainly on the locking screws. The stability of the local fracture segment being enhanced by additional BSs on the condition of axial loading is difficult to support. Furthermore, there is a potential risk of nail damage occurring when the drilling of the BS holes and thread‐cutting while placing the BS using a freehand technique. Clinically, only one BS may be used on the side with a fracture. For this study, medial and lateral displacements were tested to recreate a severely unstable fracture model. Therefore, the two BSs in the ML direction were inserted to increase BIC stability.

### Conclusions

Based on the results of this mechanical study, we conclude that both the traditional and novel BS techniques increased the primary stability of distal metaphyseal fractures. They exhibited similar results in mechanical tests. However, the novel screw helped alleviate the difficulty of tuning of the BS during operation. The time spent inserting the new BS was significantly shorter than that of the traditional one, and the damage to the nail in the novel group was more subtle than that of the traditional one. Additionally, the obvious advantage of the novel BS is that the distance and pressure between it and the IM nail can be adjusted by turning the screw, which decreases the operation time and avoids the occurrence of new fractures. These advantages provide more benefits for the clinical application of BSs.

## Declaration

None of the authors of this paper have a financial or personal relationship with other people or organizations that could inappropriately influence or bias the content of the paper.
